# Temporal dynamics of thermal warming in brown trout hepatocyte spheroids: ultrastructural and immunocytochemical evidence of cellular remodelling

**DOI:** 10.1007/s00441-026-04089-y

**Published:** 2026-07-14

**Authors:** Rodrigo Alves, Fernanda Malhão, Célia Lopes, Eduardo Rocha, Tânia Vieira Madureira

**Affiliations:** 1 Laboratory of Histology and Embryology, Department of Microscopy, ICBAS – School of Medicine and Biomedical Sciences, University of Porto, Porto, Portugal; 2 Group of Animal Morphology and Toxicology, CIIMAR/CIMAR – Interdisciplinary Centre for Marine and Environmental Research, University of Porto, Matosinhos, Portugal

**Keywords:** Primary hepatocyte spheroids, *Salmo trutta*, Thermal stress, Autophagy, Ultrastructure

## Abstract

Temperature is a key environmental driver of hepatic physiology in ectotherms, and three-dimensional (3D) fish liver models may serve as an ethically advantageous platform to investigate warming effects under controlled conditions while reducing the need for experimental animals. Despite their increasing use in toxicology, their application to assess climate-relevant temperature effects on liver function remains limited. This study investigated the temporal effects of a warming scenario on primary hepatocyte spheroids from juvenile brown trout (*Salmo trutta*), a bioindicator species. The study explores how a + 3 °C increase can affect spheroid development and maintenance, as well as its impact on metabolic activity, cell proliferation and death, and morphology over time. Spheroids were maintained at 18 °C and 21 °C for 25 days and analysed at five time points using metabolic, morphometric, immunocytochemical, and ultrastructural approaches. Mitochondrial metabolic activity, assessed by resazurin reduction, showed no significant temperature-related differences. In contrast, warming accelerated spheroid formation and produced larger spheroids. Proliferative activity, assessed by proliferating cell nuclear antigen (PCNA) immunostaining, was significantly reduced at 21 °C, while caspase-3 levels remained unchanged, indicating no increase in apoptosis. The autophagy marker microtubule-associated protein 1A/1B-light chain 3 (LC3A/B) showed lower immunoreactivity at 21 °C, with no temporal variation. Ultrastructural analysis revealed preserved hepatocyte integrity at both temperatures and abundant cytoplasmic dense bodies consistent with autolysosomal structures, which increased over time. Overall, a realistic warming scenario altered growth dynamics and cellular morphology. Further, this data reinforces that 3D fish liver models are viable alternative systems for assessing climate-driven effects.

## Introduction

The liver plays a central role in xenobiotic metabolism and is therefore widely used to assess exposure effects and mechanisms in toxicological assays (Losser and Payen 1996). In line with the 3Rs (Replacement, Reduction and Refinement) principles, in vitro liver models have emerged as well-established alternatives to animal experimentation (Louis-Maerten et al. 2024). Primary hepatocyte cultures closely reproduce in vivo liver metabolic, enzymatic, and transport functions, and offer a robust platform for mechanistic and dose–response studies, enabling multiple experimental replicates from a single biological source (Olsavsky et al. 2007; Bischof et al. 2016). Furthermore, three-dimensional (3D) hepatocyte spheroids better reproduce in vivo-like cell–cell interactions, polarity and metabolic competence than conventional two-dimensional (2D) monolayer cultures (Kammerer 2021; Holgersson et al. 2024). Recent evidence also highlights the potential of fish 3D culture systems as alternatives to in vivo studies and models for environmental research (Ranasinghe et al. 2025).

Temperature changes are recognised as major causes of liver damage (Martínez et al. 2020; Liu et al. 2022). Studies in mammalian hepatocytes showed that high temperatures disrupt metabolic and cellular functions, including glycogen metabolism, protein synthesis, oxidative balance and xenobiotic biotransformation (Schamhart et al. 1984; Mitev et al. 2005; Li et al. 2012). Heat stress may also induce heat-shock protein expression, caspase activation and reduced cell viability (Li et al. 2012; Thompson et al. 2014). The mammalian systems have allowed discrimination of general cellular heat responses; however, as endothermic organisms, they maintain internal thermal stability, limiting their ecological relevance for studying the effects of environmental warming. In contrast, ectothermic organisms such as most fish are particularly suitable models for investigating climate-driven warming, because their body temperature and cellular physiology are directly influenced by environmental temperature fluctuations (Volkoff and Rønnestad 2020; Currie and York 2026).

Fish exhibited temperature-sensitive hepatic responses, particularly regarding enzyme activity, even within ecologically relevant temperature ranges. For example, in blue tilapia (*Oreochromis aureus*), acclimatization to warmer temperatures decreased the activity of glycolytic enzymes, while lactate dehydrogenase (LDH) activity increased (Younis 2015). Similarly, in striped eel catfish (*Plotosus lineatus*) at 30 °C, glucose levels increased, accompanied by decreases in glycolysis enzyme activities and LDH activity (dos Santos Carvalho and Fernandes 2019). Additionally, hyperthermia induces oxidative stress, as evidenced by alterations in superoxide dismutase (SOD) (Liu et al. 2016) and catalase (CAT) (Lushchak and Bagnyukova 2006) activity in fish liver. Heat stress at 29 °C was also strongly correlated with oxidative damage, as noted in pikeperch (*Sander lucioperca*) liver, where total antioxidant capacity and mRNA levels of antioxidant genes were altered compared with the control group maintained at 23 °C (Wang et al. 2019). Moreover, temperature had a strong effect on temazepam biotransformation in European perch (*Perca fluviatilis*) and dragonfly larvae (*Sympetrum* sp.), with the production and subsequent accumulation of its metabolite being higher at 20 °C than at 10 °C (Cerveny et al. 2021). Similarly, in liver microsomes of Senegalese sole (*Solea senegalensis*), uridine diphosphate glucuronosyltransferase (UDPGT), 3-cyano-7-ethoxycoumarin O-deethylase (CECOD), 7-ethoxyresorufin O-deethylase (EROD) and carboxylesterase (CbE) were influenced by temperature, showing lower activities at higher temperatures (Solé et al. 2015). High-temperature stress has been associated with fish liver ultrastructural changes, including the appearance of electron-dense lipid droplets, increased lipid accumulation, reduced glycogen content, poorly developed endoplasmic reticulum with loss of its ribosomes, irregular mitochondrial morphology, and organelle disruption (Braunbeck et al. 1987; Liu et al. 2016).

Despite the growing knowledge of thermal effects on fish liver physiology, studies using 3D hepatic fish models remain scarce, even though these models have emerged as valuable platforms for studying environmentally relevant stressors and their underlying mechanisms. For example, rainbow trout liver spheroids (RTL-W1) exposed to a 5 °C increase in temperature showed reduced expression of genes involved in xenobiotic metabolism and transport, together with ultrastructural alterations of the endoplasmic reticulum and intracellular dense bodies (Esteves et al. 2025).

Within this context, the present study builds on a previously established and validated baseline characterization of primary brown trout hepatocyte spheroids (Pereira et al. 2022; Alves et al. 2023, 2024) to investigate the temporal effects of a climate change–relevant warming scenario (Soto 2018; Borgwardt et al. 2020). Spheroids were maintained at 18 °C (control) or 21 °C (+ 3 °C warming) for 25 days, and ultrastructural and immunocytochemical responses were assessed over time. This approach elucidates temperature‑induced cellular remodelling processes in a fish liver spheroid system, thereby contributing to the understanding of how moderate warming reshapes hepatic homeostasis.

## Materials and methods

### Fish

Juvenile brown trout (*Salmo trutta*), approximately one year old, were acclimated for a period of four weeks after their transport from the Aquaculture Station of Torno, Amarante, Portugal (Madureira et al. 2019). The fish (*n* = 6) exhibited a mean body weight of 62.2 ± 10.4 g and a total length of 18.3 ± 1.5 cm (mean ± standard deviation). A controlled 12 h light/12 h dark photoperiod was applied, and fish were fed daily with a commercial diet (Trout Plus 4, AquaSoja), except during the 24 h fasting period before hepatocyte isolation. Water quality parameters were monitored weekly using commercial test kits (Prodac, Cittadella, Italy) and maintained at the following levels (mean ± standard deviation): total ammonia nitrogen—0.0 ± 0.0 mg/L, nitrate—31.3 ± 2.3 mg/L, nitrite—0.04 ± 0.02 mg/L. A portable probe (DO210, VWR International, Leuven, Belgium) was used to measure dissolved oxygen (89.1 ± 1.2%) and temperature (18.3 ± 1.1 °C). pH values (7.9 ± 0.1) were determined using a pH meter (WTW pH530, Oberbayern, Germany).

### Hepatocyte isolation

Fish were euthanised with an overdose of ethylene glycol monophenyl ether (8.07291.1000, Merck KGaA, Darmstadt, Germany) at a concentration of 0.6 mL/L. No experimental procedures were conducted before euthanasia, which was performed by a certified researcher in accordance with the Portuguese Decree-Law No. 113/2013 (transposing Directive 2010/63/EU on the protection of animals for scientific purposes).

Hepatocytes were isolated through a two-step collagenase perfusion protocol previously adapted for brown trout (Madureira et al. 2015). Hepatocyte viability was assessed using an automated cell counter (Countess™, Invitrogen™, California, CA, USA) with a 1:1 dilution of the cell suspension in 0.4% trypan blue (T10282, Invitrogen™), yielding a mean viability of 91.3% ± 1.8 (mean ± SD).

### 3D cultures

Three fish per temperature (18 °C and 21 °C) were used to isolate primary hepatocytes. Cells were cultured using supplemented HyClone™ Dulbecco’s Modified Eagle Medium/Nutrient Mixture F-12 (DMEM/F-12; SH40007.01, Cytiva, MA, USA), as previously optimised for primary brown trout spheroids (Pereira et al. 2022). The supplementation included 10% charcoal-stripped fetal bovine serum (FBS; F6765, Merck KGaA, Darmstadt, Germany), 15 mM 2-[4-(2-hydroxyethyl)−1-piperazinyl] ethanesulfonic acid (HEPES, H3784, Merck KGaA, Darmstadt, Germany), and 10 mL/L antibiotic–antimycotic solution (100 ×; A2213, Merck KGaA, Darmstadt, Germany) containing 10,000 units/mL penicillin, 10 mg/mL streptomycin, and 25 μg/mL amphotericin B. Cells were seeded in non–tissue culture-treated sterile 6-well plates (351146, Falcon, Corning, New York, NY, USA) at a density of 5 × 10^5^ cells/mL, with a final volume of 3 mL per well. For each experiment, one plate was used per temperature. Right after isolation, hepatocytes were maintained either at 18 °C or 21 °C without external O₂/CO₂ supplementation, under continuous orbital agitation (~ 100 rpm) using a digital microtiter shaker (MTS 2/4, IKA^®^, Staufen, Germany). Medium was partially renewed every other day by replacing 1.5 mL with fresh medium. Sampling was conducted on days 8, 12, 16, 18 and 25 post-isolation. At each sampling time point, individual spheroids were collected using a P100 micropipette and allocated to the respective predetermined endpoints. In each assay, wells corresponding to each designated sampling day were randomly assigned on the plate to avoid bias.

### AlamarBlue™ HS assay

The alamarBlue™ HS Cell Viability Reagent (A50101, Invitrogen™ by Thermo Fisher Scientific, Eugene, OR, USA) was used to assess spheroid metabolic activity over time. In each experiment, six spheroids per sampling day were transferred to the wells of a 96-well non–tissue culture-treated plate (351172; Falcon, Corning, New York, NY, USA). Next, each well received 90 µL of fresh medium along with 10 µL of alamarBlue™ reagent. Background controls (*n* = 6 per sampling day) were prepared using fresh medium without spheroids. After 3 and 6 h of incubation with alamarBlue™, at 18 °C or 21 °C, fluorescence was measured with a Biotek Synergy™ HTX multimode microplate reader (Agilent, Santa Clara, CA, USA) using the Gen5 software (version 3.05.11). The excitation and emission wavelengths were set to 550 nm and 588 nm, respectively. Background fluorescence was subtracted from sample values.

### Spheroid biometric analysis

Spheroid images were obtained using an Olympus CKX41 light microscope (Tokyo, Japan) equipped with a Pixelink M5C-CYL-PL-D685CU digital camera (Barrington, NJ, USA) and a 10 × objective lens. For each sampling day, 30 spheroid images were taken and subsequently analysed with an open-source AnaSP software (version 2.0) to determine spheroid equivalent diameter, area, and sphericity, following established procedures (Piccinini 2015; Pereira et al. 2022).

### Immunocytochemistry

At each sampling day, six spheroids were transferred into 1.5 mL microtubes and fixed at room temperature with 500 µL of 10% neutral buffered formalin (5701, Epredia, Breda, The Netherlands). After 24 h, the fixative was replaced with 70% ethanol. Spheroids were then embedded in HistoGel^®^ (HG-4000; Epredia, Breda, The Netherlands) and processed using a 12 h routine histological protocol in an automated tissue processor (TP 1020; Leica Biosystems, Wetzlar, Germany), involving sequential dehydration through an ascending ethanol series, followed by intermediate clearing in xylene and final paraffin infiltration using two paraffin baths prior to embedding (1 h per step). Samples were embedded in paraffin (Histoplast; Epredia, Breda, The Netherlands) using an embedding station (EG1140C; Leica Biosystems, Wetzlar, Germany). Paraffin blocks (2 spheroids/sampling day/experiment) were sectioned at 3 μm (5 sections per slide) with a fully automated rotary microtome (RM2255; Leica Biosystems, Wetzlar, Germany).

For each immunocytochemical procedure (Proliferating Cell Nuclear Antigen—PCNA; cysteine-aspartic acid protease 3—caspase-3; and microtubule-associated protein 1A/1B-light chain 3—LC3A/B antibodies), slides from 2 spheroids/sampling day/experiment were selected. Slides were first deparaffinised with xylene (2 × 10 min) and hydrated in a sequence of decreasing ethanol concentrations (100%, 95%, and 70%) and water. The antigen retrieval step for PCNA was performed with tris(hydroxymethyl)aminomethane (Tris)/ethylenediaminetetraacetic acid (EDTA) buffer at pH 9.0, with 0.05% of Tween 20 (P1379, Merck, Darmstadt, Germany), preheated in a microwave until boiling. The slides were placed in the buffer for 15 min (700 W). For caspase-3 and LC3A/B, the antigen retrieval step was performed in citrate buffer (pH 6.0) preheated in a pressure cooker until boiling. The slides were then immersed, and the pressure cooker was closed. After it reached maximum pressure, 3 min were counted. Then, for both antigen retrieval protocols, the slides were allowed to cool at room temperature and washed with distilled water. A freshly prepared solution of 3% hydrogen peroxide (1.07210, Merck, Darmstadt, Germany) in methanol (32213, Honeywell, Charlotte, NC, USA) was used to block the endogenous peroxidase by immersing the slides for 10 min. The Novolink™ Max Polymer Detection System kit (Leica Biosystems, Wetzlar, Germany) was used following the manufacturer’s instructions for the following protocol steps.

Antibody incubation lasted 2 h (at ± 20 °C) in a humidified chamber, using either a mouse monoclonal anti-PCNA — clone PC10 (sc-56, Santa Cruz Biotechnology, Santa Cruz, CA), a polyclonal rabbit anti-active caspase-3 (ab4051, Abcam, Cambridge, UK) or a rabbit monoclonal anti-LC3A/B — clone D3U4C (12741, Cell Signaling Technology, Massachusetts, USA) antibodies with a dilution of 1:3000, 1:200 and 1:600 respectively, in phosphate-buffered saline (PBS) with 5% bovine serum albumin (BSA; MB04602, NZYTech, Lisbon, Portugal). PCNA, caspase-3 and LC3 are highly conserved proteins (Warbrick et al. 1998; Bell and Megeney 2017; North et al. 2025), and the antibodies have been previously used in fish samples with consistent results (Dezfuli et al. 2012; Hu et al. 2022; Valenzuela et al. 2023; de Barros et al. 2026). Liver histological sections from an adult female brown trout were used as positive controls, whereas negative controls were incubated just in PBS containing 5% BSA (MB04602, NZYTech, Lisbon, Portugal). A 3,3′-diaminobenzidine (DAB) solution was used as a chromogen, and the slides were counterstained with Mayer’s hematoxylin (1.09249.2500, Merck KGaA, Darmstadt, Germany) for 1 min. The slides were dehydrated in ethanol, cleared in xylene (28975.325, VWR  Chemicals, Fontenay-sous-Bois, France), mounted with Q Path^®^ Coverquick 2000 media (05547530, VWR Chemicals, Fontenay-sous-Bois, France) and observed in a light microscope (BX50, Olympus, Tokyo, Japan) equipped with a digital camera (EP50, Olympus, Tokyo, Japan).

The relative immunostained area (%) was quantified by differential point counting on digital images. Two spheroids were analysed per sampling day for each experiment using ImageJ (Version 1.54). A tiered point-counting grid with a 1:5 area ratio was employed: a dense grid (one point per 10,000 pixels^2^) was used to count hits on immunopositive areas, while a coarse grid (one point per 50,000 pixels^2^) was used to count hits within the entire area of the cross-sectioned spheroid. The relative immunostained area was then calculated as follows:

$$Relative\;Immunostained\;Area\;\left(\%\right)=\frac{\mathrm\Sigma\;\mathrm{Points}\;\mathrm{over}\;\mathrm{immunostained}\;\mathrm{area}}{\mathrm\Sigma\;\mathrm{Points}\;\mathrm{over}\;\mathrm{spheroid}\times5}\times100$$

### Transmission Electron Microscopy (TEM)

A pool of spheroids (3 spheroids/sampling day/experiment) was collected into microcentrifuge tubes and centrifuged (239 g, 5 min). After removing the supernatant, the samples were fixed with a freshly prepared 2.5% glutaraldehyde (Merck, Darmstadt, Germany) in sodium cacodylate-HCl buffer (0.1 M, pH 7.2) for 2 h at 4 °C.

Following this step, the fixative was washed off twice for 10 min each with the same 0.1 M cacodylate buffer (8.20670, Merck, Darmstadt, Germany). The spheroids were post-fixed in 1% osmium tetroxide (AGR1024, Agar Scientific, Essex, UK) in the same buffer for 2 h at 4 °C. Then, spheroids underwent two 10 min washes in 0.1 M cacodylate buffer, followed by dehydration. The dehydration procedure comprised 30 min passages with increasing ethanol concentrations (50%, 70%, 96%, and 100%) at 4 °C, followed by two 15 min baths of propylene oxide (8.07027.1000, Merck, Darmstadt, Germany). For 1 h at each step, the spheroids were progressively infiltrated with epoxy resin by immersing them in increasing concentrations of epoxy resin diluted in propylene oxide (1:3, 1:1, and 3:1, respectively), followed by a final solution containing only epoxy resin. Next, the spheroids were placed at 60 °C for 10 min to evaporate propylene oxide residues before being transferred to the rubber embedding moulds. For the formation of hardened blocks, the spheroids were placed in freshly prepared 100% epoxy resin-filled rubber embedding moulds and incubated for 48 h at 60 °C to enable polymerisation.

After manual razor blade trimming of the hardened blocks, the semithin sections were cut with diamond knives (Diatome, Nidau, Switzerland) on an ultramicrotome (EMUC7, Leica Biosystems, Vienna, Austria). Semithin 1.25 µm sections were collected on adhesive glass microscope slides (VWR, Leuven, Belgium) and stained for 5 min with a 1:1 mixture of 1% methylene blue (1.15943, Merck, Darmstadt, Germany) and 1% azure II (1.09211, Merck, Darmstadt, Germany) at 60 °C. The slides were washed with distilled water, dried at 60 °C and mounted with Coverquick 2000 (05547530, VWR Chemicals, Fontenay-sous-Bois, France). The slides were observed and photographed using a microscope (BX50, Olympus, Tokyo, Japan) coupled to a digital camera (EP50, Olympus, Tokyo, Japan). Ultrathin sections (90 nm) were obtained using an ultramicrotome (EMUC7, Leica Biosystems, Vienna, Austria) with diamond knives (Diatome, Nidau, Switzerland) and adhered to 200 square mesh copper grids (Electron Microscopy Sciences, Hatfield, PA, USA). Grids were contrasted with 3% aqueous uranyl acetate (Merck KGaA, Darmstadt, Germany) (20 min) and Reynold’s lead citrate made from lead nitrate and sodium citrate (Merck KGaA, Darmstadt, Germany) (10 min) (Reynolds 1963).

A transmission electron microscope (100CXII, JEOL, Tokyo, Japan) operated at 60 kV was used to observe the grids, and a digital camera (Orius SC1000 CCD, Gatan, Pleasanton, CA, USA) was used to capture the images.

### Statistical analyses

Data were analysed using Past software (version 3.25) (Hammer 2001), and graphs were generated with GraphPad Prism (version 8.0.1). Group means were compared using two-way analysis of variance (two-way ANOVA) followed by Tukey’s post-hoc test. Prior to ANOVA, data were assessed for normality (Shapiro–Wilk test) and homogeneity of variance (Levene’s test). All data met the assumptions. Statistical significance was set at *p* < 0.05.

## Results

### AlamarBlue™ HS assay

**Fig. 1:**
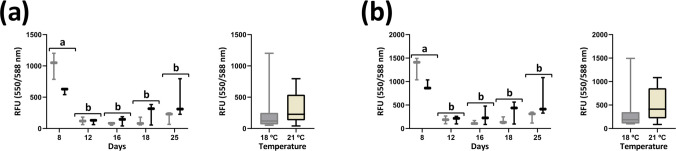
Resazurin fluorescence assay in of brown trout primary hepatocyte spheroids cultured for 8, 12, 16, 18 and 25 days, at 18 °C and 21 °C, after 3 h (**a**) and 6 h (**b**) incubation (*n* = 6 spheroids/day/fish). Fluorescence values expressed as relative fluorescence units (RFU, 550/588 nm) are plotted over days in culture. Data are shown as median, minimum, maximum, and 25th and 75th percentiles. Different lowercase letters indicate significant differences (*p* < 0.05) between culture days

Resazurin fluorescence levels in primary brown trout hepatocyte spheroids cultured at 18 and 21 °C showed similar temporal patterns both after 3 h and 6 h of incubation (Fig. 1). A two-way ANOVA was performed to evaluate the effects of culture days and temperature on the resazurin metabolisation after 6 h of incubation. There was a significant main effect for culture days (*F*(4, 20) = 11.60, *p* < 0.001), but no significant main effect of temperature (*F*(1, 20) = 2.64, *p* = 0.120), and no significant interaction (*F*(4, 20) = 1.07, *p* = 0.398). Fluorescence levels were significantly higher on day 8 (median at 18 °C – 1050 RFU; median at 21 °C – 629 RFU) compared to the remaining culture days. The values were stable from day 12 (median at 18 °C – 119 RFU; median at 21 °C – 131 RFU) to day 25 (median at 18 °C – 230 RFU; median at 21 °C – 310 RFU). Although the overall ANOVA did not detect a significant effect of temperature, median fluorescence on day 8 was numerically lower at 21 °C than at 18 °C. Overall, an opposite trend was observed in the remaining days, with higher levels recorded at 21 °C than at 18 °C, particularly from day 16 to day 25.

### Spheroids biometry

**Fig. 2:**
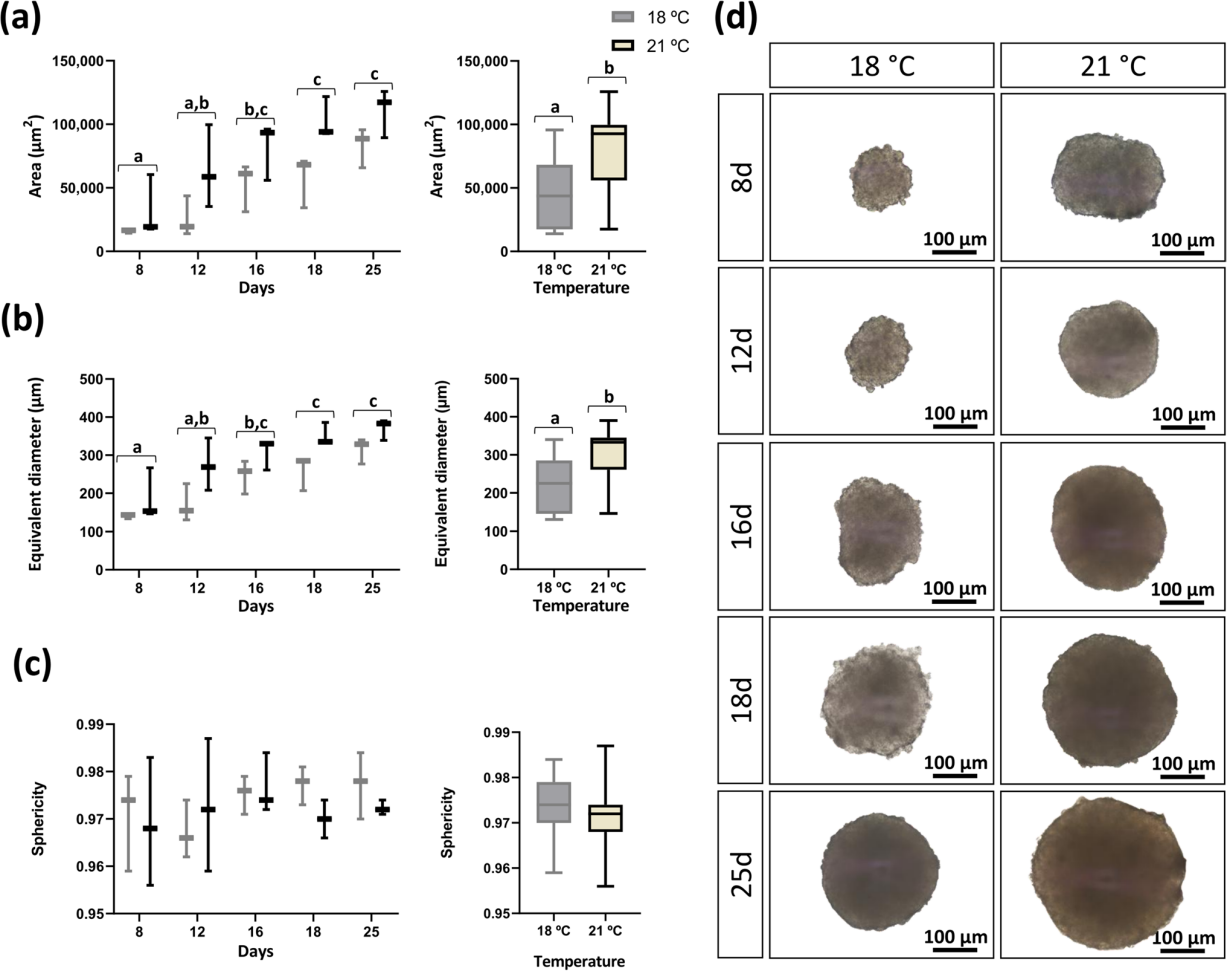
Biometric parameters of brown trout primary hepatocyte spheroids cultured for 8, 12, 16, 18, and 25 days, at 18 °C and 21 °C (*n* = 30 spheroids/day/fish), showing (**a**) area, (**b**) equivalent diameter, and (**c**) sphericity, along with representative bright-field images (**d**) at different culture days (3 fish/temperature). Data are shown as median, minimum, maximum, and 25th and 75th percentiles. Different lowercase letters indicate significant differences (*p* < 0.05) between culture days or between temperatures

The morphometric characterisation of brown trout primary hepatocyte spheroids maintained at 18 °C and 21 °C throughout the 25 days in culture is presented in Fig. 2. A two-way ANOVA was performed to evaluate the effects of culture days and temperature on the area, equivalent diameter and sphericity of primary hepatocyte spheroids.

The area results showed a significant main effect for culture days (*F*(4, 20) = 12.21, *p* =  < 0.001), a significant main effect for temperature (*F*(1, 20) = 18.14, *p* =  < 0.001), and a non-significant interaction between culture days and temperature (*F*(4, 20) = 0.453, *p* = 0.769). Overall, the area of spheroids stabilised after the 12th day in culture (median value at 18 °C – 19,493 µm^2^; median value at 21 °C – 58,640 µm^2^) and remained stable until the 25th day (median value at 18 °C – 88,669 µm^2^; median value at 21 °C − 117,287 µm^2^) (Fig. 2a).

Equivalent diameter statistics indicated a significant main effect for culture days (*F*(4, 20) = 14.59, *p* =  < 0.001); a significant main effect for temperature (*F*(1, 20) = 19.22, *p* =  < 0.001); and a non-significant interaction between culture days and temperature (*F*(4, 20) = 0.44, *p* = 0.781). The equivalent diameter also stabilised after day 12 (median value at 18 °C – 155 µm; median value at 21 °C – 269 µm). Over time, cells progressively aggregated into compact spheroids, which increased in diameter mainly during the early culture days (Fig. 2d). At 21 °C, spheroids began to aggregate earlier than those maintained at 18 °C. Sphericity ranged from 0.956 to 0.987, and no significant changes were detected between culture days (*F*(4, 20) = 0.71, *p* = 0.592) or temperatures (*F*(1, 20) = 0.24, *p* = 0.628). The interaction between the two parameters was not significant (*F*(4, 20) = 0.57, *p* = 0.689).

### Immunocytochemistry

**Fig. 3:**
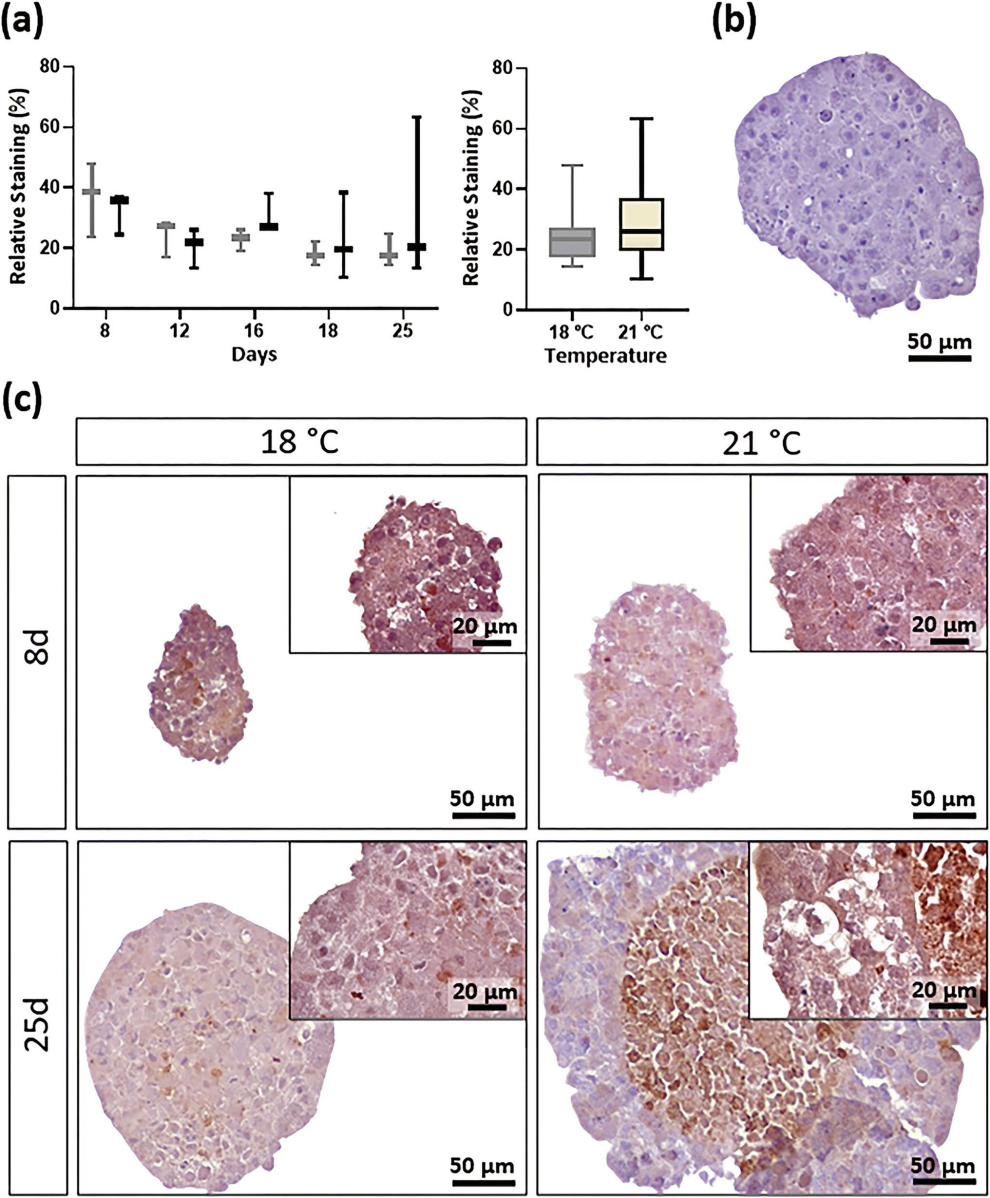
Caspase-3 relative immunostained area (%) (**a**) (*n* = 2 spheroids/day/fish). Representative images of the negative control (**b**) and brown trout primary hepatocyte spheroids cultured for 8 and 25 days at 18 °C and 21 °C (**c**), showing positive immunostaining characterised by a diffuse brown cytoplasmic immunolabelling pattern distributed throughout the spheroids. (**c**) day 25 at 21 °C represents an example of necrotic core formation, with strong caspase-3 immunolabelling. Graphical data (**a**) are shown as median, minimum, maximum, and 25th and 75th percentiles

The caspase-3 relative immunostained areas (%) and the corresponding immunolabelling patterns of brown trout primary hepatocyte spheroids at 18 °C and 21 °C, over 25 days, are presented in Fig. 3. Caspase-3 immunostaining was cytoplasmic and diffusely distributed throughout the spheroids. No immunostaining was observed in the negative controls (Fig. 3b). A two-way ANOVA was performed to evaluate the effects of culture days and temperature on the relative percentage of caspase-3 immunopositive areas. Results showed a non-significant effect for culture days (*F*(4, 20) = 1.88, *p* = 0.154); non-significant effect for temperature (*F*(1, 20) = 0.35, *p* = 0.559); and a non-significant interaction between culture days and temperature (*F*(4, 20) = 0.492, *p* = 0.741). Notably, on day 25 at 21 °C, immunostaining was predominantly localised in the spheroid's central core (Fig. 3c). However, the observed pattern was restricted to a few spheroids at 21 °C.

**Fig. 4:**
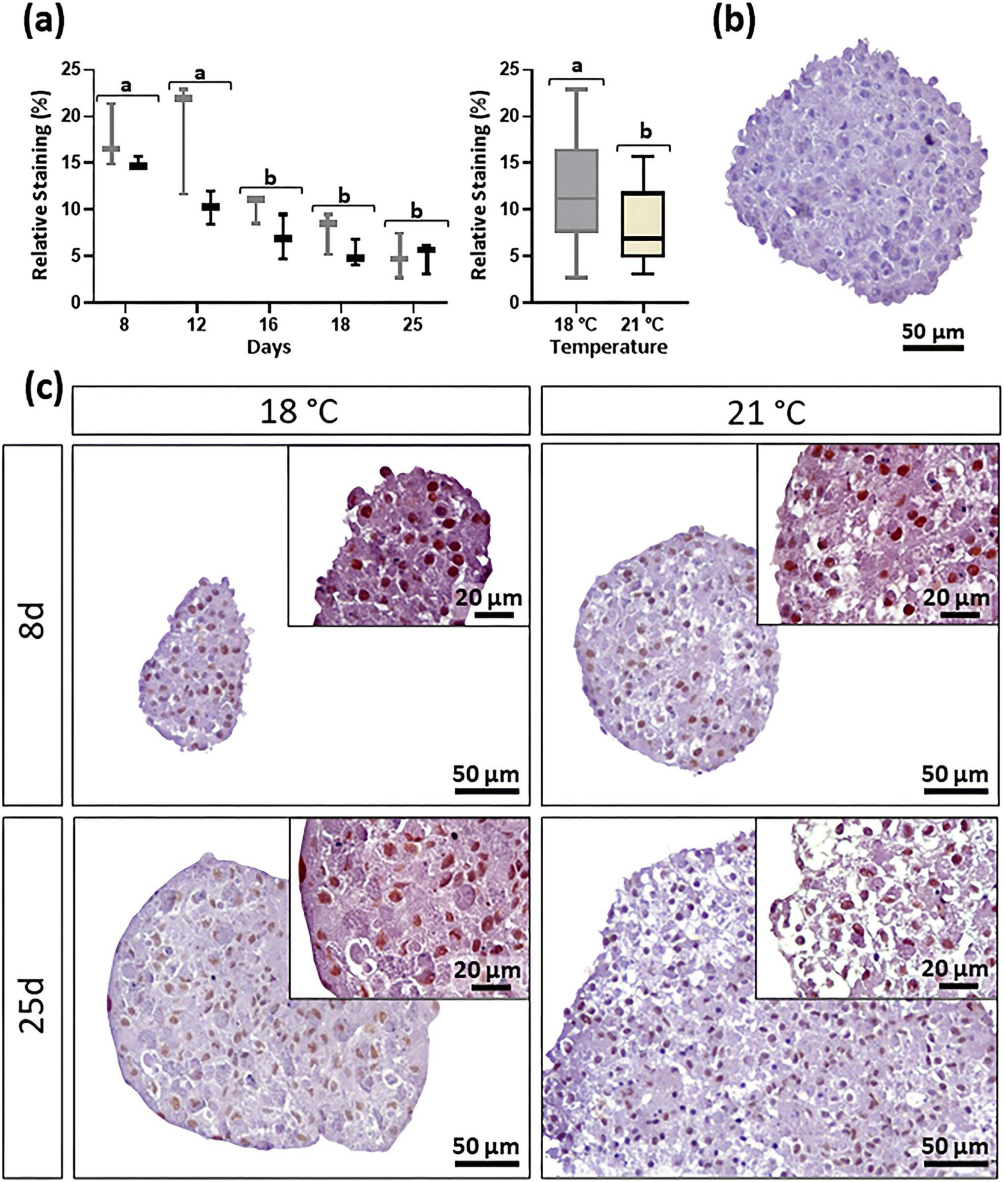
Proliferating cell nuclear antigen (PCNA) relative immunostained area (%) (**a**) (*n* = 2 spheroids/day/fish). Representative images of the negative control (**b**) and brown trout primary hepatocyte spheroids cultured for 8 and 25 days at 18 °C and 21 °C (**c**), illustrating the nuclear immunolabelling patterns distributed throughout the spheroids. Positive immunostaining appears brown. Graphical data (**a**) are shown as median, minimum, maximum, and 25th and 75th percentiles. Different lowercase letters indicate significant differences (*p* < 0.05) between culture days or between temperatures

Figure 4 shows the PCNA relative immunostained areas (%) along with the immunolabelling patterns of brown trout primary hepatocyte spheroids maintained at 18 °C and 21 °C for 25 days in culture. The PCNA immunostaining was exclusively nuclear (Fig. 4c). No immunostaining was detected in the negative controls (Fig. 4b). A two-way ANOVA was performed to evaluate the effects of culture days and temperature on the relative percentage of PCNA immunopositive areas. Results showed a significant effect for culture days (*F*(4, 20) = 19.15, *p* =  < 0.001); significant effect for temperature (*F*(1, 20) = 11.30, *p* = 0.003); and a non-significant interaction between culture days and temperature (*F*(4, 20) = 1.93, *p* = 0.143). At both temperatures, the relative percentage of PCNA immunopositive areas was significantly higher at days 8 (median value at 18 °C – 17%; median value at 21 °C – 15%) and 12 (median value at 18 °C – 22%; median value at 21 °C – 10%) compared with the remaining days. After day 16, values stabilized until the end of the culture period (Fig. 4a). Overall, the percentage of PCNA immunopositive areas was significantly lower in spheroids cultured at 21 °C (median value on days 8, 12, 16, 18 and 25 − 15%, 10%, 7%, 5% and 6%, respectively) compared with those maintained at 18 °C (median value on days 8, 12, 16, 18 and 25 − 17%, 22%, 11%, 9% and 5%, respectively) (Fig. 4a).

**Fig. 5:**
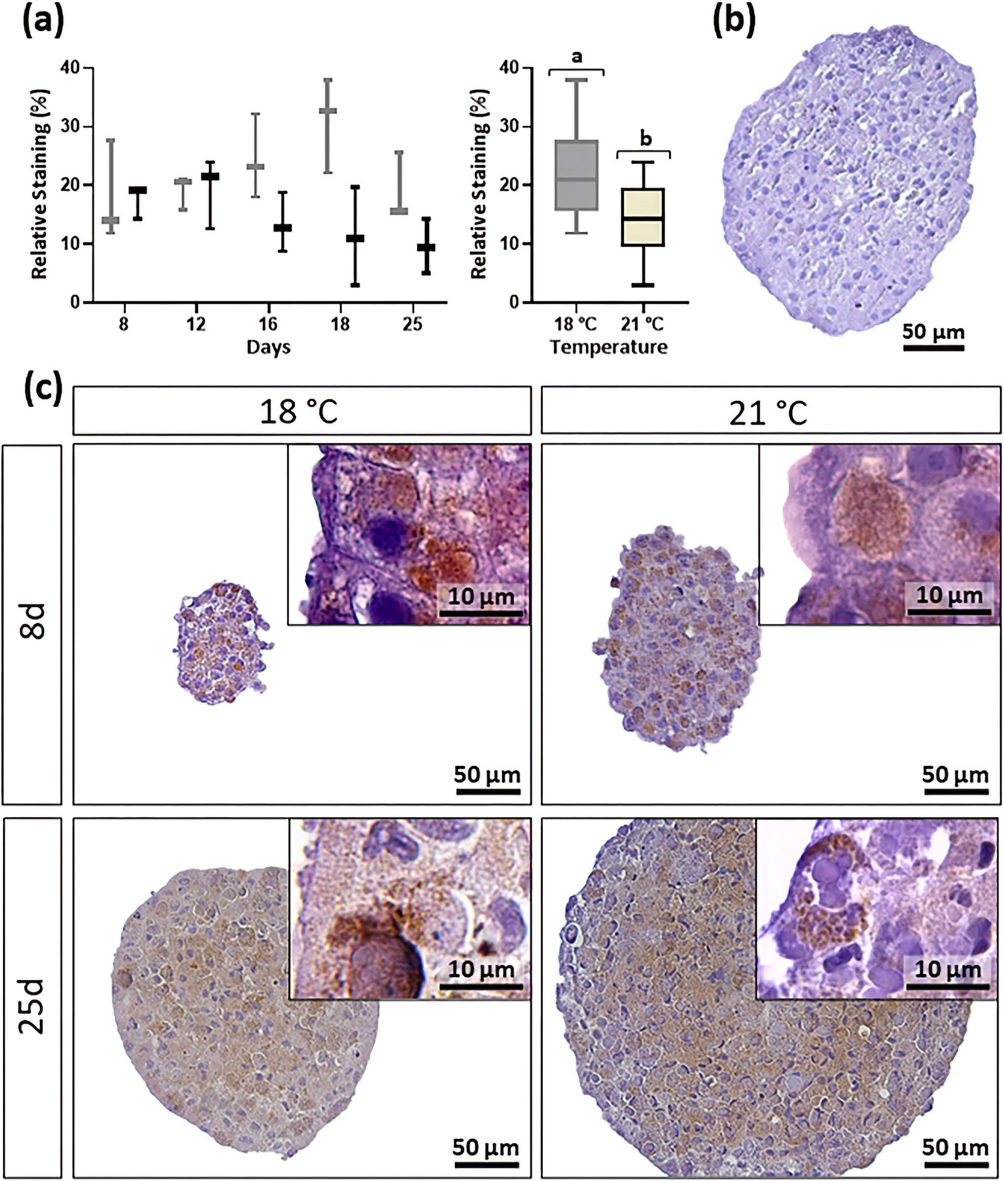
Microtubule-associated protein 1A/1B-light chain 3 (LC3A/B) relative immunostained area (%) (**a**) (*n* = 2 spheroids/day/fish). Representative images of the negative control (**b**) and brown trout primary hepatocyte spheroids cultured for 8 and 25 days at 18 °C and 21 °C (**c**), illustrating the diffuse cytoplasmic immunolabelling or dots distributed throughout the spheroids. Positive immunostaining appears brown. Graphical data (**a**) are shown as median, minimum, maximum, and 25th and 75th percentiles. Different lowercase letters indicate significant differences (*p* < 0.05) between temperatures

Figure 5 presents the LC3A/B relative immunostained areas (%) and the immunolabelling patterns of primary hepatocyte spheroids from brown trout cultured for 25 days at 18 °C and 21 °C. The LC3A/B immunostaining was diffuse cytoplasmic or presented dots (puncta) distributed throughout the spheroid. No immunolabelling was evident in the negative controls (Fig. 5b). A two-way ANOVA was performed to evaluate the effects of culture days and temperature on the relative immunopositive area (%) of LC3A/B in primary hepatocyte spheroids. Data showed a non-significant effect for culture days (*F*(4, 19) = 0.92, *p* = 0.471); a significant main effect for temperature (*F*(1, 19) = 11.51, *p* = 0.003); and a non-significant interaction between culture days and temperature (*F*(4, 19) = 2.54, *p* = 0.074). At 21 °C, the relative immunopositive areas of LC3A/B on days 16 (median value − 13%), 18 (median value − 11%), and 25 (median value − 9%) were significantly lower than at 18 °C (median value on days 16, 18 and 25 − 23%, 33% and 16%, respectively) (Fig. 5a).

### Spheroid morphology in semithin sections

**Fig. 6:**
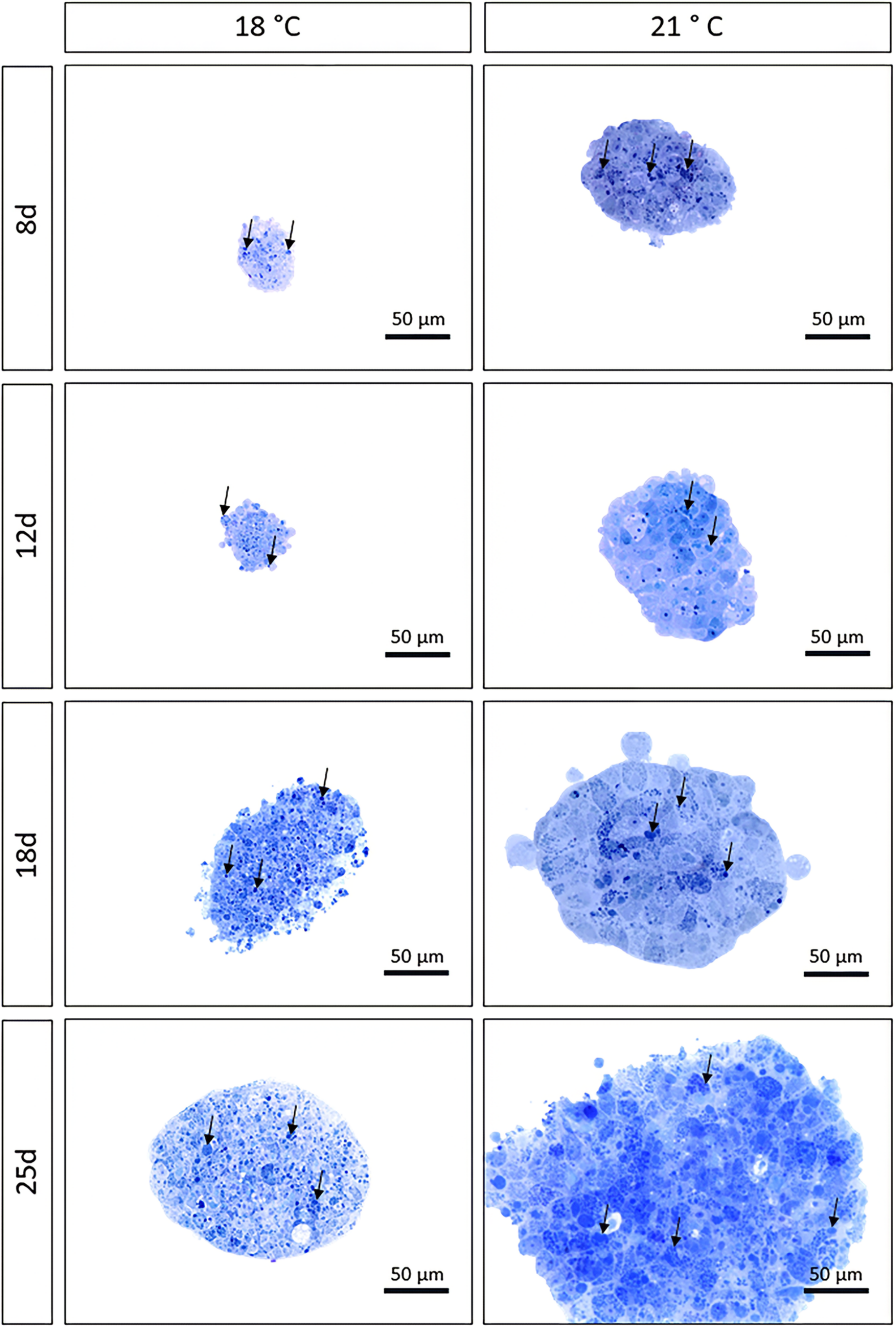
Representative methylene blue and azure-stained semithin sections of brown trout primary hepatocyte spheroids cultured for 8, 12, 18 and 25 days, at 18 °C and 21 °C (*n* = 3 spheroids/day/fish). Black arrows point to examples of dense cytoplasmic structures of varying sizes and densities

Semithin sections of brown trout primary hepatocyte spheroids cultured at 18 °C and 21 °C revealed that, at both temperatures, roundish to slightly elliptical compact spheroids were formed. Spheroids formed under 18 °C were smaller than those at 21 °C. This size difference was first observed at day 8 and persisted throughout the culture period until day 25. Across all spheroids, regardless of temperature or culture day, a prominent morphological feature was the presence of dense cytoplasmic structures that varied in number, size, and density (Fig. 6).

### TEM morphological characterization

**Fig. 7:**
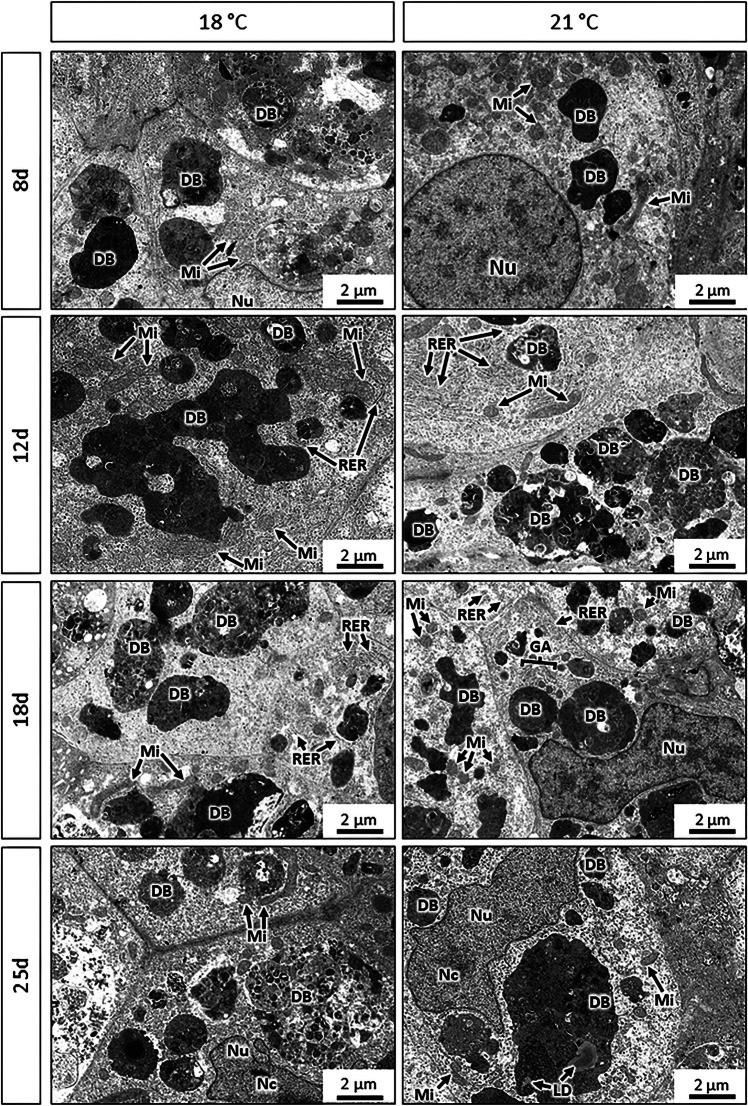
Representative transmission electron microscopy (TEM) images of brown trout primary hepatocyte spheroids cultured for 8, 12, 18 and 25 days, at 18 °C and 21 °C (*n* = 3 spheroids/day/fish). DB: dense bodies, GA: Golgi apparatus, LD: lipid droplet, Mi: mitochondria, Nu: nucleus, Nc: nucleolus, RER: rough endoplasmic reticulum

Ultrastructure analysis of brown trout primary hepatocyte spheroids showed that the cells were tightly packed and exhibited round to irregularly shaped euchromatic nuclei, some with prominent nucleoli and others displaying sparse areas of coarse chromatin (Fig. 7). Overall, the cytoplasmic organelle content seemed similar at both temperatures (18 and 21 °C), consisting mainly of rough endoplasmic reticulum (RER) cisterns (isolated or arranged in a few parallel rows), free ribosomes, and round to elongated mitochondria (Mi). Golgi apparatuses (GA) and lipid droplets (LD) were also sporadically observed. LD were frequently associated with more developed dense bodies (DB), particularly on day 25 (e.g., Fig. 7).

**Fig. 8:**
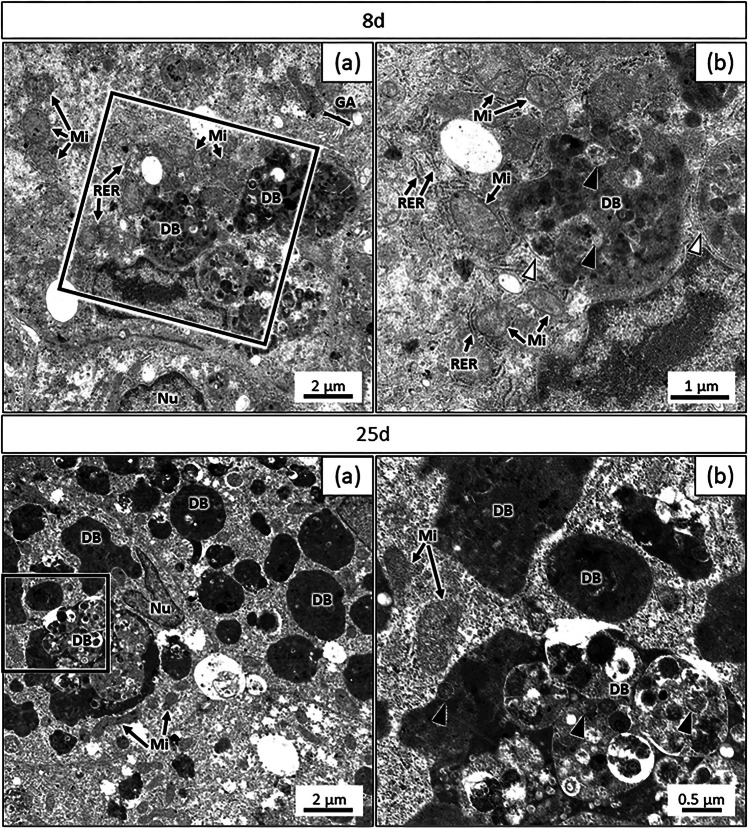
Representative transmission electron microscopy (TEM) images of brown trout primary hepatocyte spheroids cultured for 8 and 25 days, at 18 °C (**a** and **b**) (*n* = 3 spheroids/day/fish). Panel **b** shows higher magnifications of the areas indicated in panel **a**. DB: dense bodies, GA: Golgi apparatus, Mi: mitochondria; Nu: nucleus, RER: rough endoplasmic reticulum. Black arrowheads indicate mitochondria enclosed within DB, whereas white arrowheads highlight the membranes surrounding some DB

**Fig. 9:**
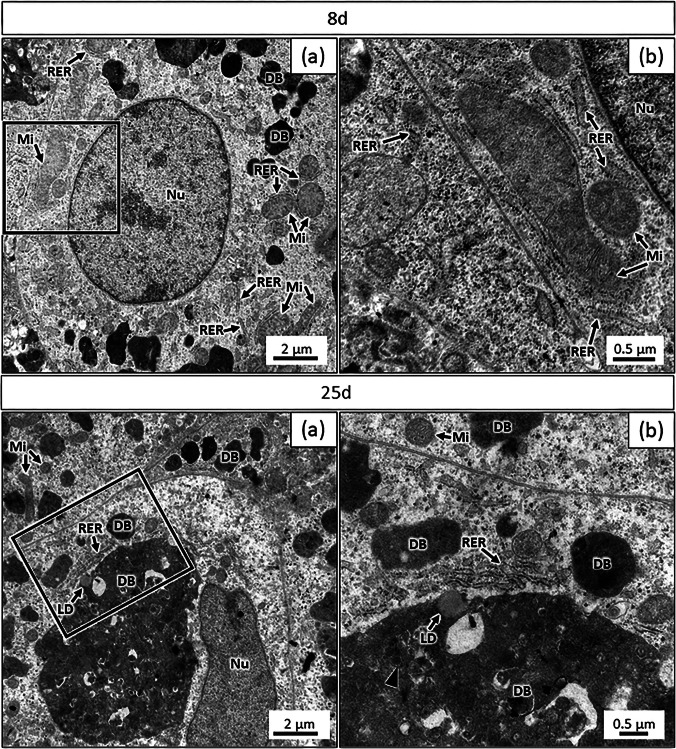
Representative transmission electron microscopy (TEM) images of brown trout primary hepatocyte spheroids cultured for 8 and 25 days, at 21 °C (**a** and **b**) (*n* = 3 spheroids/day/fish). Panel **b** shows higher magnifications of the areas indicated in panel **a**. DB: dense bodies, LD: lipid droplets, Mi: mitochondria; Nu: nucleus, RER: rough endoplasmic reticulum. Black arrowheads indicate mitochondria enclosed within DB

**Fig. 10:**
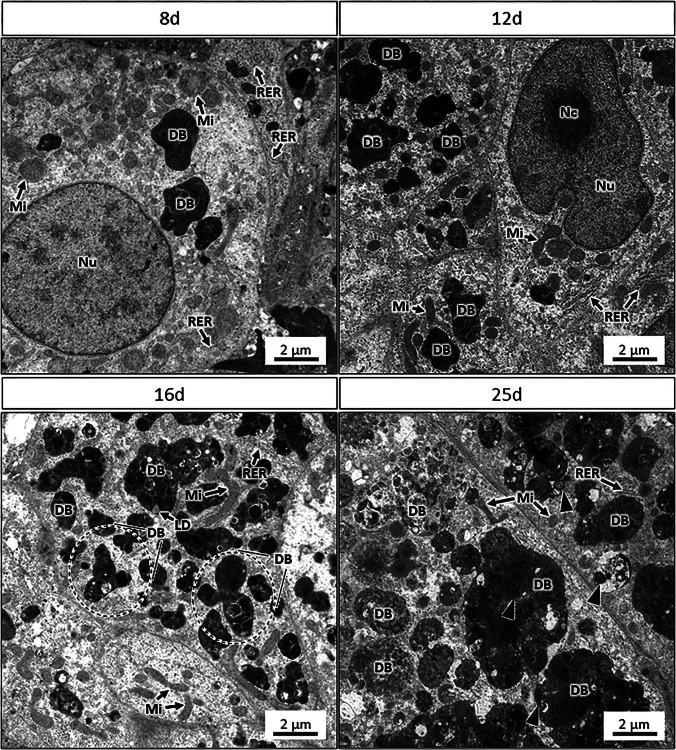
Representative transmission electron microscopy (TEM) images of brown trout primary hepatocyte spheroids cultured for 8, 12, 16 and 25 days, at 18 and 21 °C (*n* = 3 spheroids/day/fish). DB: dense bodies, LD: lipid droplets, Mi: mitochondria; Nu: nucleus, Nc: nucleolus, RER: rough endoplasmic reticulum. Dashed circles highlight the coalescence of smaller DB into larger structures over the course of culture, while black arrowheads indicate mitochondria enclosed within DB

A notable ultrastructural feature was the presence of DB, already detectable in semithin sections, which varied in size and developmental stage and were evident from the first sampling day (day 8). Both the number and size of DB increased progressively throughout the culture period. DB were present, in different proportions, in nearly all hepatocytes within the spheroids at both temperatures, with no distinct spatial distribution pattern. A more detailed description of DB development over time is provided in Figs. 8, 9 and 10.

At both temperatures, as culture time progressed, DB at different developmental stages gradually occupied the cytoplasm. In some cases, DB contained membrane structures — putative organelle debris derived from autophagy — which were clearly visible within the DB and typically displayed a concentric lamellar appearance. In some DB, intact mitochondria were still visible, as shown by the black arrowheads in Figs. 8 and 9. Other DB showed much more electron density, making it harder to see trapped organelles and membrane boundaries. It was observed that some DB were surrounded by a single membrane, but in others, especially in highly electron‑dense profiles, the membrane contour could not be clearly resolved.

In Fig. 8, it is possible to observe that, for spheroids cultured at 18 °C, at day 8, DB were less electron-dense than those on day 25. At this temperature, spheroids on day 25 had two distinct areas within DB: a lower-density area, where concentric structures containing cell debris and mitochondria were visible, and a higher-density area at the DB periphery.

A very similar cytoplasmic pattern was observed in spheroids cultured at 21 °C, on the same sampling days (days 8 and 25), as shown in Fig. 9. At 25 days in culture, in addition to DB occupying a significant portion of the cytoplasm, numerous well-preserved organelles were still visible at both temperatures.

The DB tended to increase in number and size over time in culture, in part due to the confluence of two or more DB, as highlighted by the dashed circle in Fig. 10. This figure also shows different aspects of the DB morphology: some DB display concentric lamellar structures containing cell debris, while others are more electron-dense or have mixed areas of both morphologies.

## Discussion

Brown trout liver spheroids derived from juvenile fish have previously been established and validated as an alternative in vitro model for assessing the effects of different hormones (Pereira et al. 2022; Alves et al. 2023, 2024). Based on the stabilisation of biometry, morphology and basal gene expression profiles, spheroids between days 12 and 20 have been identified as the optimal experimental window for screening hepatic adaptive and toxicological responses to xenobiotics (Alves et al. 2023). When maintained at 18 °C, this model showed robustness and high reproducibility, with consistent biometric parameters and viability. Those parameters were also confirmed in the present study. For example, by day 16 of culture, spheroids typically exhibited a median equivalent diameter of approximately 200 µm and high structural integrity, as indicated by a median sphericity exceeding 0.9 (Pereira et al. 2022; Alves et al. 2023, 2024).

In the context of ongoing global warming, it is increasingly important to establish a baseline understanding of spheroid performance at higher temperatures, given the potential for interaction effects between rising temperatures and exposure to environmental contaminants. Despite this, knowledge of the influence of temperature increases on fish spheroid growth kinetics is limited. The present study addresses this gap by simulating a 3 °C increase in culture temperature (18 °C versus 21 °C) and assessing temperature-dependent effects over time. Spheroids were collected at days 8, 12, 16, 18, and 25 of culture at both temperatures. To the best of our knowledge, this study is the first to investigate temporal dynamics alongside temperature effects in fish primary hepatocyte spheroids.

Overall, the increase in culture temperature was not associated with statistically significant differences in cellular metabolic activity, as estimated by the resazurin assay, which primarily reflects mitochondrial activity. Nevertheless, after an initial stress adaptation, spheroids at 21 °C tended to exhibit higher metabolic activity than those at 18 °C. Regarding sampling days, spheroids sampled at day 8 showed significantly higher metabolic activity than those sampled at later sampling points, at both temperatures. This pattern may reflect the early post-isolation phase, during which cells retain a higher metabolic vigour and more closely resemble the physiological state of primary hepatocytes in vivo, before progressively adapting to long-term 3D culture conditions. Supporting this interpretation, caspase-3 immunostaining remained relatively stable across temperatures and sampling days, suggesting that the lower metabolic activity observed at later stages was not necessarily associated with increased apoptotic activity.

Nonetheless, spheroids cultured at 21 °C showed a faster kinetic formation rate, becoming established earlier than those maintained at 18 °C. From day 8 onwards, and across all sampling days, spheroids at 21 °C were larger, with higher areas and equivalent diameters than those at 18 °C, while sphericity remained unaffected. The larger spheroid size did not translate into an increase in caspase-3 immunostaining signal. Observations of a necrotic core at 21 °C (e.g., Fig. 3, day 25 at 21 °C) were rare and appeared to be qualitatively associated with increased spheroid size rather than temperature per se, based on visual inspection rather than a systematic analysis of spheroid size–necrosis relationships. Although reactive oxygen species (ROS) production and oxidative stress markers are commonly used to assess cellular damage, these endpoints were not considered within the scope of the present study.

To further characterise spheroid growth kinetics, cell proliferation was assessed in the present study through immunocytochemical quantification of PCNA. The latter is a highly conserved protein commonly used as a cell proliferation marker, as it is predominantly expressed during the S phase of the cell cycle (Ortego et al. 1995). During the first 12 days, spheroids at both temperatures showed high proliferative activity, whereas from day 16 onward, PCNA immunostaining declined and stabilised until the last sampling day (day 25). This temporal pattern corroborates the idea that, after isolation, spheroids undergo an initial proliferative phase before transitioning to a mature and stable state after day 12, as previously described for this model (Alves et al. 2023). Spheroids cultured at 21 °C showed lower PCNA immunostaining than those maintained at 18 °C. This output aligns with reports of reduced PCNA expression in mouse hepatocytes under heat stress conditions at higher temperatures (44 or 46 °C). In the present study, the decreased PCNA expression at 21 °C was not accompanied by increased hepatocyte necrosis or apoptosis (Li et al. 2012), suggesting that it basically reflects altered proliferative dynamics rather than cytotoxicity.

The ultrastructural analysis by TEM not only complemented the previous morphological characterisation of brown trout primary hepatocyte spheroids maintained in culture for up to 25 days, but also disclosed no marked differences between spheroids at 18 °C and 21 °C. Irrespective of the temperature, most hepatocytes remained intact throughout the culture period, with well-preserved organelles. The organelle content included mitochondria of different sizes and morphologies, rough endoplasmic reticulum cisternae, an underdeveloped Golgi apparatus (suggestive of a low secretory state), and lipid droplets, which is consistent with previous descriptions of brown trout hepatocytes (Rocha et al. 1994). A consistent feature observed in spheroids cultured at both temperatures was the abundance of DB, whose size and number increased over the culture period. Morphologically, these structures ranged from electron-dense to mixed areas of electron density, interspersed with concentric lamellar structures, residual cell debris, and remaining organelles and lipid droplets. These aspects are consistent with autophagic activity, a lysosome-mediated degradation pathway that plays a central role in cellular clearance, targeting long-lived macromolecular complexes and organelles to maintain intracellular homeostasis (Jung et al. 2019; Liu et al. 2023). Autophagy has also been associated with cell remodelling (Saera-Vila et al. 2016) and mechanisms underlying regeneration and repair across different regenerative contexts (Moreno-Blas et al. 2025), processes that are vital for maintaining the viability of spheroids cultured for 25 days.

In the most well-characterised form of autophagy, macroautophagy, a double-membrane autophagosome fuses with a lysosome, forming an autolysosome, a single membrane vesicle that enables nonspecific degradation of cytoplasmic components (Jung et al. 2019; De Mazière et al. 2022). DB were observed in the present study, either surrounded by a single membrane or with undefined membrane limits (likely due to the very dense periphery of the organelle). Spheroids' autophagy likely represents an adaptive remodelling response to the culture conditions. We hypothesised that this process is initiated shortly after the isolation procedure, in line with reports that stated that autophagy is activated within hours in cell culture (Ni et al. 2011). By day 8 onwards, the majority of the observed DB are likely autolysosomes actively engaged in enzymatic degradation, contributing to intracellular homeostasis and the maturation of the spheroids.

The DB also revealed aspects of mitophagy (mitochondrial digestion) (Chen et al. 2022; Xia and Wang 2023) and lipophagy (lipid droplet digestion) (Wang et al. 2018). Both processes have already been described in fish and are often associated with adaptive responses to nutrient deprivation. Protein degradation via autophagy is an essential adaptive mechanism in fish, enabling the breakdown of macromolecules to generate metabolites required for catabolic and anabolic processes and facilitating responses to various environmental stressors (Kawsar et al. 2025). Similar DB, although smaller and less abundant, have been described in the rainbow trout liver cell line RTL-W1 (Malhão et al. 2013; Holgersson et al. 2024), suggesting that DB are a conserved feature of hepatocyte/liver cell homeostasis across different in vitro fish models.

In this study, the ultrastructural evidence of autophagy was complemented by immunocytochemical analysis of the well-established autophagy marker LC3. LC3, with its two most studied isoforms, LC3A and LC3B, undergoes the conversion from the cytosolic LC3-I form to the lipidated, membrane-associated LC3-II, which localises autophagosomes during their formation and maturation (Koukourakis et al. 2015). LC3-I manifests as a diffuse cytoplasmic signal, representing the soluble, unconjugated form, while the LC3-II (lipidated form), integrated into autophagosome membranes, presents as distinct punctate spots (dots) within the cytoplasm, making puncta a key indicator of active autophagy (Zhang et al. 2016). Autophagy is a dynamic process in which LC3-II associated with the outer autophagosome membrane is removed by a cysteine protease, while the LC3-II on the inner membrane is maintained until the autophagosome fuses with lysosomes and its contents are degraded (Mizushima et al. 2010). The antibody used in this study recognises both LC3A and LC3B (I and II), so the observed immunostaining should reflect the total LC3 pool involved in the autophagic activity.

Strong LC3 immunostaining was observed at both temperatures, corroborating the ultrastructural findings that demonstrated that autophagy was present in all spheroids. Overall, spheroids cultured at 21 °C showed lower LC3 immunoreactivity than those maintained at 18 °C. Nonetheless, TEM revealed DB consistent with autolysosomes at both temperatures, indicating that the reduced LC3 signal at 21 °C is unlikely to reflect autophagy suppression. Instead, the faster spheroid formation kinetics observed at 21 °C may be consistent with a hypothetical accelerated autophagic flux, in which LC3-II is more rapidly degraded following autophagosome–lysosome fusion. Such a mechanism could help to explain the reduced LC3 availability for immunostaining, although alternative explanations cannot be ruled out, and autophagic flux was not directly assessed in this study. Furthermore, no significant differences in LC3A/B immunostaining were detected among spheroids sampled throughout the 25-day culture period. This pattern is compatible with the notion that, from day 8 to day 25, hepatocytes had reached a steady state in which autophagosome formation was approximately balanced by its degradation. According to Plaza-Zabala et al. (2021), basal autophagy reflects such a balance, which may have been reached before day 8. If this was the case, our interpretation would be that the results of this study are consistent with a stable autophagic plateau rather than demonstrating a change in autophagic flux per se.

## Conclusion

In summary, this study revealed that a 3 °C increase in temperature influences the kinetics and morphology of primary brown trout hepatocyte spheroids by accelerating spheroid formation and increasing spheroid diameter. Although the resazurin assay and caspase-3 immunocytochemistry did not indicate marked temperature-related alterations in metabolic activity or apoptosis under the present experimental conditions, the resazurin data suggested a trend towards higher metabolic activity in spheroids cultured at 21 °C after the initial adaptation period. Moreover, spheroids cultured at 21 °C showed lower PCNA and LC3 immunostaining, suggesting reduced proliferative activity and temperature modulation of autophagy-related markers. Ultrastructural analyses confirmed long-term hepatocyte integrity and highlighted autophagy as a key adaptive mechanism supporting spheroid viability. Taken together, these findings support the applicability of fish liver spheroids as a relevant and robust in vitro model to evaluate temperature-mediated effects on fish hepatic physiology and cellular homeostasis, with direct relevance for climate‑warming scenarios. Given this context, future studies should address how elevated temperatures, alone and in combination with chemical stressors, modulate hepatocyte responses in this model system and further characterise autophagic flux using complementary approaches.

## Data Availability

All data supporting the findings of this study are available within the paper.

## References

[CR1] Alves RF, Lopes C, Rocha E, Madureira TV (2023) A step forward in the characterization of primary brown trout hepatocytic spheroids as experimental models. Animals (Basel) 13(14):2277. 10.3390/ani13142277 37508054 10.3390/ani13142277 PMC10376616

[CR2] Alves RF, Lopes C, Rocha E, Madureira TV (2024) Estrogenic responsiveness of brown trout primary hepatocyte spheroids to environmental levels of 17α-ethinylestradiol. J Xenobiot 14(3):1064–1078 39189175 10.3390/jox14030060 PMC11348032

[CR3] Bell RA, Megeney LA (2017) Evolution of caspase-mediated cell death and differentiation: twins separated at birth. Cell Death Differ 24(8):1359–1368 28338655 10.1038/cdd.2017.37 PMC5520454

[CR4] Bischof I, Köster J, Segner H, Schlechtriem C (2016) Hepatocytes as in vitro test system to investigate metabolite patterns of pesticides in farmed rainbow trout and common carp: comparison between in vivo and in vitro and across species. Comp Biochem Physiol C Toxicol Pharmacol 187:62–73 27185525 10.1016/j.cbpc.2016.05.003

[CR5] Borgwardt F, Unfer G, Auer S, Waldner K, El-Matbouli M, Bechter T (2020) Direct and indirect climate change impacts on brown trout in central Europe: how thermal regimes reinforce physiological stress and support the emergence of diseases. Front Environ Sci 8:59

[CR6] Braunbeck T, Gorgas K, Storch V, Völkl A (1987) Ultrastructure of hepatocytes in golden ide (*Leuciscus idus melanotus L*; *Cyprinidae*:*Teleostei*) during thermal adaptation. Anat Embryol (Berl) 175(3):303–313 3826656 10.1007/BF00309844

[CR7] Cerveny D, Fick J, Klaminder J, McCallum ES, Bertram MG, Castillo NA, Brodin T (2021) Water temperature affects the biotransformation and accumulation of a psychoactive pharmaceutical and its metabolite in aquatic organisms. Environ Int 155:106705 34139590 10.1016/j.envint.2021.106705

[CR8] Chen H, Zhao F, Chen K, Guo Y, Liang Y, Zhao H, Chen S (2022) Exposure of zebrafish to a cold environment triggered cellular autophagy in zebrafish liver. J Fish Dis 45(7):991–1000 35395109 10.1111/jfd.13620

[CR9] Currie S, York JM (2026) Temperature sensing in fishes: mechanisms and modulation in a warming world. J Exp Biol 229(Suppl_1):jeb250889 10.1242/jeb.250889 41668661

[CR10] de Barros A, Santos D, Lourenço T, Lopes C, Madureira TV, Rocha E (2026) Seasonal and sex-specific liver plasticity in brown trout: Estrogen-responsive targets and cell turnover dynamics. Animals 16(7):1073 41976051 10.3390/ani16071073 PMC13072334

[CR11] De Mazière A, van der Beek J, van Dijk S, de Heus C, Reggiori F, Koike M, Klumperman J (2022) An optimized protocol for immuno-electron microscopy of endogenous LC3. Autophagy 18(12):3004–3022 35387562 10.1080/15548627.2022.2056864 PMC9673964

[CR12] Dezfuli BS, Giari L, Lui A, Squerzanti S, Castaldelli G, Shinn AP, Manera M, Lorenzoni M (2012) Proliferative cell nuclear antigen (PCNA) expression in the intestine of *Salmo trutta trutta* naturally infected with an acanthocephalan. Parasit Vectors 5(1):198 22967751 10.1186/1756-3305-5-198 PMC3583471

[CR13] dos Santos Carvalho C, Fernandes MN (2019) Effects of copper toxicity at different pH and temperatures on the in vitro enzyme activity in blood and liver of fish. Prochilodus Lineatus Mol Biol Rep 46(5):4933–4942 31264160 10.1007/s11033-019-04944-y

[CR14] Esteves T, Malhão F, Rocha E, Lopes C (2025) Effects of benzo[k]fluoranthene at two temperatures on viability, structure, and detoxification-related genes in rainbow trout RTL-W1 cell spheroids. Toxics 13(4):302 40278618 10.3390/toxics13040302 PMC12031258

[CR15] Hammer O (2001) PAST: paleontological statistics software package for education and data analysis. Palaeontol Electron 4:9

[CR16] Holgersson V, Joyce S, Brookman-Amissah M, Lammel T (2024) Comparative analysis of 3D and 2D *in vitro* models of the permanent fish liver cell line RTL-W1: metabolic capabilities and responses to xenobiotics. Ecotoxicol Environ Saf 288:117327 39550873 10.1016/j.ecoenv.2024.117327

[CR17] Hu B, Lelek S, Spanjaard B, El-Sammak H, Simoes MG, Mintcheva J, Aliee H, Schaefer R, Meyer AM, Theis F (2022) Origin and function of activated fibroblast states during zebrafish heart regeneration. Nat Genet 54(8):1227–1237 35864193 10.1038/s41588-022-01129-5 PMC7613248

[CR18] Jung M, Choi H, Mun JY (2019) The autophagy research in electron microscopy. Appl Microsc 49(1):11 33580401 10.1186/s42649-019-0012-6 PMC7809580

[CR19] Kammerer S (2021) Three-dimensional liver culture systems to maintain primary hepatic properties for toxicological analysis in vitro. Int J Mol Sci 22(19):10214 34638555 10.3390/ijms221910214 PMC8508724

[CR20] Kawsar MA, Adikari D, Zhang Y (2025) Autophagy in aquatic animals: Mechanisms, implications, and future directions. Front Immunol 16:1612178 40529357 10.3389/fimmu.2025.1612178 PMC12170313

[CR21] Koukourakis MI, Kalamida D, Giatromanolaki A, Zois CE, Sivridis E, Pouliliou S, Mitrakas A, Gatter KC, Harris AL (2015) Autophagosome proteins LC3A, LC3B and LC3C have distinct subcellular distribution kinetics and expression in cancer cell lines. PLoS ONE 10(9):e0137675 26378792 10.1371/journal.pone.0137675 PMC4574774

[CR22] Li SQ, Li RF, Xi SM, Hu S, Jia ZQ, Li SP, Wen XL, Song YK, Li S, Li SP (2012) Systematical analysis of impacts of heat stress on the proliferation, apoptosis and metabolism of mouse hepatocyte. J Physiol Sci 62(1):29–43 22125186 10.1007/s12576-011-0183-6 PMC10717989

[CR23] Liu B, Xu P, Brown PB, Xie J, Ge X, Miao L, Zhou Q, Ren M, Pan L (2016) The effect of hyperthermia on liver histology, oxidative stress and disease resistance of the Wuchang bream, *Megalobrama amblycephala*. Fish Shellfish Immunol 52:317–324 27016402 10.1016/j.fsi.2016.03.018

[CR24] Liu E, Zhao X, Li C, Wang Y, Li L, Zhu H, Ling Q (2022) Effects of acute heat stress on liver damage, apoptosis and inflammation of pikeperch (*Sander lucioperca*). J Therm Biol 106:103251 35636889 10.1016/j.jtherbio.2022.103251

[CR25] Liu S, Yao S, Yang H, Liu S, Wang Y (2023) Autophagy: regulator of cell death. Cell Death Dis 14(10):648 37794028 10.1038/s41419-023-06154-8 PMC10551038

[CR26] Losser MR, Payen D (1996) Mechanisms of liver damage. Semin Liver Dis 16(4):357–367 9027949 10.1055/s-2007-1007249

[CR27] Louis-Maerten E, Rodriguez Perez C, Cajiga RM, Persson K, Elger BS (2024) Conceptual foundations for a clarified meaning of the 3Rs principles in animal experimentation. Anim Welf 33:e37 39347486 10.1017/awf.2024.39 PMC11428052

[CR28] Lushchak VI, Bagnyukova TV (2006) Temperature increase results in oxidative stress in goldfish tissues 2 antioxidant and associated enzymes. Comp Biochem Physiol C Toxicol Pharmacol 143(1):36–41 16426898 10.1016/j.cbpc.2005.11.018

[CR29] Madureira TV, Malhão F, Pinheiro I, Lopes C, Ferreira N, Urbatzka R, Castro LF, Rocha E (2015) Estrogenic and anti-estrogenic influences in cultured brown trout hepatocytes: Focus on the expression of some estrogen and peroxisomal related genes and linked phenotypic anchors. Aquat Toxicol 169:133–142 26539803 10.1016/j.aquatox.2015.10.010

[CR30] Madureira TV, Costa JL, Malhão F, Lopes C, Goncalves JF, Rocha E (2019) Design of a multi-parametric profile for assessing the acclimation period of juvenile brown trout after an acute transport to new housing environment. Appl Anim Behav Sci 219:104835

[CR31] Malhão F, Urbatzka R, Navas JM, Cruzeiro C, Monteiro R, Rocha E (2013) Cytological, immunocytochemical, ultrastructural and growth characterization of the rainbow trout liver cell line RTL-W1. Tissue Cell 45(3):159–174 23305652 10.1016/j.tice.2012.10.006

[CR32] Martínez D, Vargas-Lagos C, Saravia J, Oyarzún R, Loncoman C, Pontigo J, Vargas-Chacoff L (2020) Cellular stress responses of *Eleginops maclovinus* fish injected with *Piscirickettsia salmonis* and submitted to thermal stress. Cell Stress Chaperones 25(1):93–104 31834618 10.1007/s12192-019-01051-6 PMC6985426

[CR33] Mitev S, Dinevska-Kovkarovska S, Miova B (2005) Effect of the acclimation to high environmental temperature on the activity of hepatic glycogen phosphorylase (a+b and a), liver glycogen content and blood glucose level in rats. J Therm Biol 30(8):563–568

[CR34] Mizushima N, Yoshimori T, Levine B (2010) Methods in mammalian autophagy research. Cell 140(3):313–326 20144757 10.1016/j.cell.2010.01.028 PMC2852113

[CR35] Moreno-Blas D, Adell T, González-Estévez C (2025) Autophagy in tissue repair and regeneration. Cells 14(4):282 39996754 10.3390/cells14040282 PMC11853389

[CR36] Ni H-M, Bockus A, Wozniak AL, Jones K, Weinman S, Yin X-M, Ding W-X (2011) Dissecting the dynamic turnover of GFP-LC3 in the autolysosome. Autophagy 7(2):188–204 21107021 10.4161/auto.7.2.14181 PMC3039769

[CR37] North BJ, Fracchiolla D, Ragusa MJ, Martens S, Shoemaker CJ (2025) The rapidly expanding role of LC3-interacting regions in autophagy. J Cell Biol 224(8):e202504076 40704964 10.1083/jcb.202504076 PMC12313203

[CR38] Olsavsky KM, Page JL, Johnson MC, Zarbl H, Strom SC, Omiecinski CJ (2007) Gene expression profiling and differentiation assessment in primary human hepatocyte cultures, established hepatoma cell lines, and human liver tissues. Toxicol Appl Pharmacol 222(1):42–56 17512962 10.1016/j.taap.2007.03.032 PMC2974173

[CR39] Ortego LS, Hawkins WE, Walker WW, Krol RM, Benson WH (1995) Immunohistochemical detection of proliferating cell nuclear antigen (PCNA) in tissues of aquatic animals utilized in toxicity bioassays. Mar Environ Res 39(1–4):271–273

[CR40] Pereira IL, Lopes C, Rocha E, Madureira TV (2022) Establishing brown trout primary hepatocyte spheroids as a new alternative experimental model-testing the effects of 5α-dihydrotestosterone on lipid pathways. Aquat Toxicol 253:106331 36327687 10.1016/j.aquatox.2022.106331

[CR41] Piccinini F (2015) AnaSP: a software suite for automatic image analysis of multicellular spheroids. Comput Methods Programs Biomed 119(1):43–52 25737369 10.1016/j.cmpb.2015.02.006

[CR42] Plaza-Zabala A, Sierra-Torre V, Sierra A (2021) Assessing autophagy in microglia: a two-step model to determine autophagosome formation, degradation, and net turnover. Front Immunol 11:620602 33584716 10.3389/fimmu.2020.620602 PMC7878397

[CR43] Ranasinghe N, Lee S-S, Gamage L, Lee T-H (2025) 3D cell culture model – a substitution for future in vivo fish study? Mol Biol Rep 52(1):726 40668430 10.1007/s11033-025-10821-8

[CR44] Reynolds ES (1963) The use of lead citrate at high pH as an electron-opaque stain in electron microscopy. J Cell Biol 17(1):208 13986422 10.1083/jcb.17.1.208 PMC2106263

[CR45] Rocha E, Monteiro R, Pereira CA (1994) The liver of the brown trout, *Salmo trutta fario*: A light and electron microscope study. J Anat 185(Pt 2):241 7961130 PMC1166753

[CR46] Saera-Vila A, Kish PE, Louie KaW, Grzegorski SJ, Klionsky DJ, Kahana A (2016) Autophagy regulates cytoplasmic remodeling during cell reprogramming in a zebrafish model of muscle regeneration. Autophagy 12(10):1864–1875 27467399 10.1080/15548627.2016.1207015 PMC5066936

[CR47] Schamhart DHJ, Berendsen W, van Rijn J, van Wijk R (1984) Comparative studies of heat sensitivity of several rat hepatoma cell lines and hepatocytes in primary culture1. Cancer Res 44(10):4507–4516 6467209

[CR48] Solé M, Varó I, González-Mira A, Torreblanca A (2015) Xenobiotic metabolism modulation after long-term temperature acclimation in juveniles of *Solea senegalensis*. Mar Biol 162(2):401–412

[CR49] Soto B (2018) Climate-induced changes in river water temperature in North Iberian Peninsula. Theor Appl Climatol 133(1):101–112

[CR50] Thompson SM, Callstrom MR, Butters KA, Knudsen B, Grande JP, Roberts LR, Woodrum DA (2014) Heat stress induced cell death mechanisms in hepatocytes and hepatocellular carcinoma: in vitro and in vivo study. Lasers Surg Med 46(4):290–301 24643941 10.1002/lsm.22231 PMC4269152

[CR51] Valenzuela CA, Azúa M, Álvarez CA, Schmitt P, Ojeda N, Mercado L (2023) Evidence of the autophagic process during the fish immune response of skeletal muscle cells against *Piscirickettsia salmonis*. Animals 13(5):880 36899738 10.3390/ani13050880 PMC10000225

[CR52] Volkoff H, Rønnestad I (2020) Effects of temperature on feeding and digestive processes in fish. Temperature (Austin) 7(4):307–320 33251280 10.1080/23328940.2020.1765950 PMC7678922

[CR53] Wang J, Han SL, Li LY, Lu DL, Limbu SM, Li DL, Zhang ML, Du ZY (2018) Lipophagy is essential for lipid metabolism in fish. Sci Bull 63(879–617):882 10.1016/j.scib.2018.05.026 36658967

[CR54] Wang Y, Li C, Pan C, Liu E, Zhao X, Ling Q (2019) Alterations to transcriptomic profile, histopathology, and oxidative stress in liver of pikeperch (*Sander lucioperca*) under heat stress. Fish Shellfish Immunol 95:659–669 31706008 10.1016/j.fsi.2019.11.014

[CR55] Warbrick E, Lane DP, Glover DM, Heatherington W (1998) PCNA binding proteins in *Drosophila melanogaster*: The analysis of a conserved PCNA binding domain. Nucleic Acids Res 26(17):3925–3932 9705499 10.1093/nar/26.17.3925 PMC147798

[CR56] Xia Y, Wang W-X (2023) Bioimaging tools reveal copper processing in fish cells by mitophagy. Aquat Toxicol 261:106633 37451870 10.1016/j.aquatox.2023.106633

[CR57] Younis EM (2015) Variation in metabolic enzymatic activity in white muscle and liver of blue tilapia, *Oreochromis aureus*, in response to long-term thermal acclimatization. Chin J Oceanol Limnol 33(3):696–704

[CR58] Zhang Z, Singh R, Aschner M (2016) Methods for the detection of autophagy in mammalian cells. Curr Protoc Toxicol 69(1):20–12 10.1002/cptx.11 PMC4982470 27479363

